# Identifying Climate-Induced Groundwater Depletion in GRACE Observations

**DOI:** 10.1038/s41598-019-40155-y

**Published:** 2019-03-11

**Authors:** Brian F. Thomas, James S. Famiglietti

**Affiliations:** 1 Department of Geology and Environmental Science, University of Pittsburgh, Pittsburgh, PA 15260 USA; 2 Global Institute for Water Security, School of Environment and Sustainability, and Department of Geography and Planning, University of Saskatchewan, Saskatoon, SK S7N 0J9 Canada

## Abstract

Depletion of groundwater resources has been identified in numerous global aquifers, suggesting that extractions have exceeded natural recharge rates in critically important global freshwater supplies. Groundwater depletion has been ascribed to groundwater pumping, often ignoring influences of direct and indirect consequences of climate variability. Here, we explore relations between natural and human drivers and spatiotemporal changes in groundwater storage derived from the Gravity Recovery and Climate Experiment (GRACE) satellites using regression procedures and dominance analysis. Changes in groundwater storage are found to be influenced by direct climate variability, whereby groundwater recharge and precipitation exhibited greater influence as compared to groundwater pumping. Weak influence of groundwater pumping may be explained, in part, by quasi-equilibrium aquifer conditions that occur after “long-time” pumping, while precipitation and groundwater recharge records capture groundwater responses linked to climate-induced groundwater depletion. Evaluating groundwater response to climate variability is critical given the reliance of groundwater resources to satisfy water demands and impending changes in climate variability that may threaten future water availability.

## Introduction

The bulk of freshwater resources available for human use are stored beneath the land surface and are often tapped to satisfy agricultural and domestic water demands^1^. Globally, groundwater fulfills demands for nearly 2 billion people^2^, and provides more than 40% of water consumed for irrigation^3^. Groundwater depletion is recognized as a global phenomenon^4–7^ where continued exploitation of groundwater resources is deemed to pose major challenges to sustainable groundwater strategies. Future reliance on groundwater to fulfill water demands is questionable since many global groundwater systems are nonrenewable^8^, suggesting that modern-day recharge cannot sustain current groundwater withdrawals, while a majority of global aquifers are deemed stressed^7^, where groundwater stress is defined as a ratio of use and availability. The expectation that increased dependency on groundwater may materialize in drying areas of the world and in regions projected to exhibit decreases in groundwater recharge due to climate change^1,9^ will further exacerbate critical groundwater storage declines. Climate variability influences groundwater storage by altering groundwater recharge^10,11^ and triggering changes in groundwater use^9,12–15^. Thus, attributing variable effects of climatic and anthropogenic factors that influence groundwater storage is important in assessments of groundwater availability and vulnerability. However, direct observation of groundwater elevations and storage changes are limited in many regions due to the cost of regular data collection^16^, restraining our assessment of groundwater responses.

Remote sensing of global groundwater storage changes, with the launch of the Gravity Recovery and Climate Experiment (GRACE) satellites, has enabled important contributions to our understanding of global groundwater^1,17^. GRACE observes gravity anomalies which are converted to monthly changes in terrestrial water storage^18^. Changes in groundwater storage may be isolated employing water balance methods whereby terrestrial water storage changes result due to changes in soil moisture, snow water, and surface water stores (See Methods and Data). In this evaluation, changes in groundwater storage for the contiguous U.S. are characterized by isolating a representative groundwater storage signal from GRACE. In this study, it is assumed that GRACE-derived groundwater storage changes reflect *in-situ* groundwater responses driven by anthropogenic influences and climate variability.

Assessments of GRACE-derived groundwater storage changes have focused on single aquifer^4,5,19,20^ or large global aquifer systems^7,21^. Often, these studies characterize changes in groundwater storage as a single metric, typically the slope of groundwater storage change over time^5,6,22–24^, which fails to capture direct and indirect links between groundwater and climate^15^. Assumptions implied in groundwater storage trends are that net balance between inflows and outflows may be characterized by a single value. Thus, although a trend in groundwater storage implies a loss (or increase) of storage, changes in the distribution, timing and volume of groundwater and increases in groundwater pumping occur concurrently, complicating our interpretation of storage change drivers. Given the critical nature of groundwater to fulfill water demands^1^, it is important to differentiate factors that produce observable changes in groundwater storage. Using multiple datasets (GRACE, water use, climate) and methods (nonparametric trend analysis, ordinary least squares multivariate regression (MLR), and dominance analysis), we explore changes in groundwater storage as observed by the GRACE satellites and their relation to climate induced factors including groundwater recharge, precipitation and groundwater pumping. Our goal in this study is to identify the influence of climate via groundwater recharge and precipitation compared to human influence via groundwater use. The MLR approach is applied to enable the making of quantitative and rigorous statements regarding the influence of climatic and anthropogenic influences. Further, MLR permits application of dominance analysis, which is used to determine whether an explanatory variable is dominant over another, referred to here as strong (dominant over) or weak (not dominant over) influence.

## Results

### Trends in Groundwater Storage Anomaly Changes

**Figure 1:**
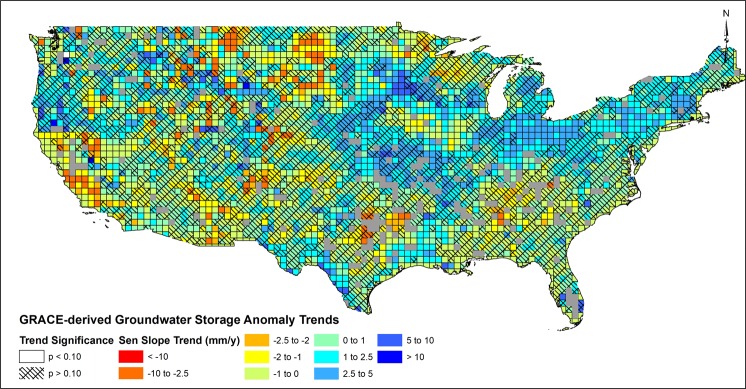
Trends in GRACE-derived groundwater storage anomalies determined by Seasonal Mann-Kendall trend tests for slope significance and Sen Slope estimator for slope magnitude. Negative trends indicate an average decline in groundwater storage while positive trends indicate an average increase in groundwater storage over the time period 10/2003 to 12/1015. Greyed squares represent regions that may be influenced by reservoirs (See Supplemental).

Trends in groundwater storage at 0.5-degree JPL GRACE mascon^25^ grids were estimated using a combined Seasonal Mann-Kendall trend test for detection of a monotonic trend significance and the Sen Slope estimator for trend magnitude^26^ (See Methods and Data). Although simplistic in their approach, nonparametric trend methods were applied to avoid violating assumptions connected to parametric trend tests^26^. Figure 1 depicts gridded trends over the period of 10/2003–12/2015, where the study period coincides with availability of U.S. Geological Survey groundwater use records^27–30^. Approximately 30% of all gridded trends exhibited significant trends [n = 1115, N = 3748, p < 0.10]. Figure 1 shows the spatial variability in groundwater trends are generally correlated to county-based US Geological Survey records of total groundwater pumping [See Supplemental]. In the Central Valley Aquifer in California, reported decreases in groundwater storage are well recognized^5,20,31,32^. Extreme trends in groundwater storage changes in the Central Valley are noted within the southern portions of the aquifer, within the Tulare River basin, consistent with previous spatial studies which depict groundwater depletion during the GRACE record^33–35^. In the Lower Missouri Basin, studies have identified increases in groundwater storage linked to flooding potential^36^ and reported increases in baseflow contributions from groundwater stores^37^. Northern portions of the High Plains Aquifer have experienced increases in groundwater storage attributed to groundwater management schemes^38^ and cropping patterns^39^. In the southern portions of the High Plains Aquifer, declines in groundwater storage are attributed to consumptive groundwater use due to crop water requirements^39^ and drought^3^. Significant decreases in groundwater storage are noted in the Northern Great Plains aquifer, an aquifer identified as unsustainable in previous studies^40^ given aquifer response to prolonged drought across the upper Great Plains. Decreases in groundwater storage in the southeastern US corroborate results of Russo and Lall^12^, where decreases in storage are attributed to combinations of groundwater use and drought. Groundwater trends in the Upper Midwest and Northeastern US have been shown to exhibit positive trends^41^, similar to results depicted in Fig. 1. Spatial patterns of groundwater trends are similar to hydrograph recession-based watershed storage trends^42^, further suggesting that general groundwater trends depicted in Fig. 1 are representative of *in-situ* groundwater storage changes.

### Influence of Groundwater Recharge

**Figure 2:**
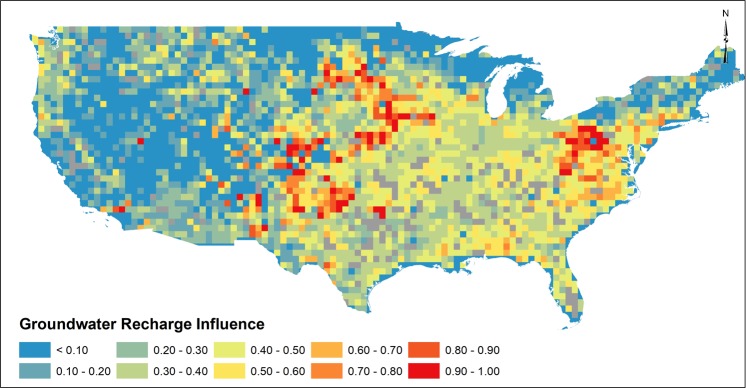
Influence of groundwater recharge to monthly GRACE-derived groundwater storage as determined by dominance analysis. Red indicates strong influence were blue represents low influence. Greyed squares represent regions that may be influenced by reservoirs.

The groundwater continuity equation is defined as the difference between inflow and outflow equaling some change in groundwater storage. Groundwater recharge represents the primary inflow to groundwater systems and is thus expected to reflect a strong influence on observed groundwater storage changes due to climate variability^14,15^. Following Li *et al*.^43^, groundwater recharge was approximated as drainage from the lowest model soil layer, often referred to as subsurface runoff, from the Variable Infiltration Capacity (VIC) land surface model^44^ within the National Land Data Assimilation System (NLDAS)^45^. Gridded approximations of groundwater recharge were used in multivariate regression (MLR) procedures (See Methods and Data) to identify relations of groundwater recharge and GRACE-derived groundwater storage changes. Influence of monthly groundwater recharge as estimated from dominance analysis identified weak influence throughout much of the arid/semi-arid western US (Fig. 2). In the western US, water limited systems, characterized by the scenario where potential evapotranspiration is greater than precipitation resulting in a system where actual evapotranspiration is controlled by precipitation, results in hydrologic budgets with little potential for diffuse groundwater recharge. In such water limited systems, groundwater recharge tends to occur during extreme precipitation events^11,46^ characterized as short duration, high intensity events. In the southwest, short duration recharge events may fail to be fully represented in monthly simulated groundwater recharge data as used in this analysis. Strong influence of groundwater recharge was noted in the Central US suggesting a higher propensity for groundwater recharge to be represented in groundwater storage changes. Depth to groundwater in the Central US was reported to often be shallow (>20 m)^47^. Shallow groundwater tables would likely be more influenced by changes in groundwater storage, given that infiltration processes may occur on shorter time-scales as compared to deep or confined aquifer systems. Further, groundwater recharge influence in the Central US is notably higher near large river system networks (Missouri and Platte Rivers) which may be an artifact of the chosen representation of groundwater recharge. Since groundwater recharge varies spatially as a result of physical attributes, including soil type, depth to groundwater, vadose zone porosity, hydrogeology and precipitation patterns, strong influence regions within the Central US are attributed to hydrogeologic characteristics combined with depth to groundwater. Low variability in groundwater recharge influence (0.4–0.6, Fig. 2) in the southeastern US is attributed to shallow groundwater and low variability in monthly precipitation. Further, the southeastern US represents energy limited systems, characterized by the scenario where potential evapotranspiration is less than precipitation thus resulting in a system where actual evapotranspiration is controlled by net radiation, which thus creates the potential for seasonally-based groundwater recharge^48^. In the northeastern US, groundwater recharge influence was weak, likely a function of high variability in hydrogeologic conditions characteristic of glacial aquifer systems.

### Influence of Precipitation

**Figure 3:**
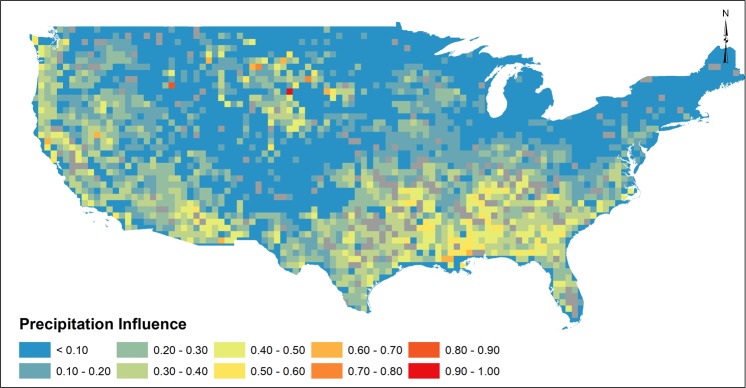
Influence of monthly precipitation to monthly GWA as determined by dominance analysis. Greyed squares represent regions that may be influenced by reservoirs.

Correlations between precipitation and groundwater have a long history in groundwater studies given the assumption that precipitation-driven recharge could be used to quantify groundwater availability^49^. Recent analyses correlated groundwater storage changes to long-term climate indices assuming long-times are necessary to link observed storage changes^12,50^. Thomas *et al*.^11^ showed that episodic events in the arid southwestern US during summer monsoons accounted for significant groundwater storage changes, similar to other arid regions^51^. Further, studies^14,52–54^ have suggested variable responses in groundwater storage due to climate variability. For observed GRACE-derived groundwater storage changes, the influence of monthly precipitation was spatially variable in the southwest (Fig. 3). Weak precipitation influence on groundwater storage may be due to the inability of episodic/extreme short-duration precipitation events to be captured in monthly precipitation records. In addition, variability in precipitation influence may be attributed to spatial distributions of shallow versus deep aquifer systems. Over the eastern US, although variability in month-to-month precipitation is low, a clear distinction in precipitation influence is noted between the northeast and southeast, where weak influence (0–0.2, Fig. 3) was observed in the northeast with strong (0.3–0.5, Fig. 3) influence observed in the southeast. This difference in precipitation influence is attributed to the heterogeneous nature of glacial aquifer systems in the northeast where previous studies have identified weak correlation between groundwater and precipitation^55^ compared to large coastal plain and surficial aquifer systems in the southeast. Regions within the Great Plains were found to exhibit strong precipitation influence, spatially coincident with the Sandhills region of Nebraska where recharge rates are noted be to high^56,57^. High precipitation influence was noted in the Lower Mississippi Valley, similar to results identified in previous studies^12^ where vector autoregressive approaches identified strong groundwater responses due to precipitation. Although patterns of influence in Fig. 3 corroborate many previous studies, our analysis is limited by the data available (GRACE) over a short-time period (10/2003–12/2015). Studies^12,13^ suggest that precipitation influx contributing to groundwater storage may take decades, whereas annual and seasonal precipitation was tested in this study. The influence of precipitation to groundwater responses ignores lag times known to exist between precipitation and groundwater storage^58^ that may not be fully captured by GRACE. In addition, low influence of precipitation in northern latitudes may be due to freezing conditions in winter months resulting in snow pack accumulation which would not be captured in monthly precipitation records.

### Influence of Pumping

**Figure 4:**
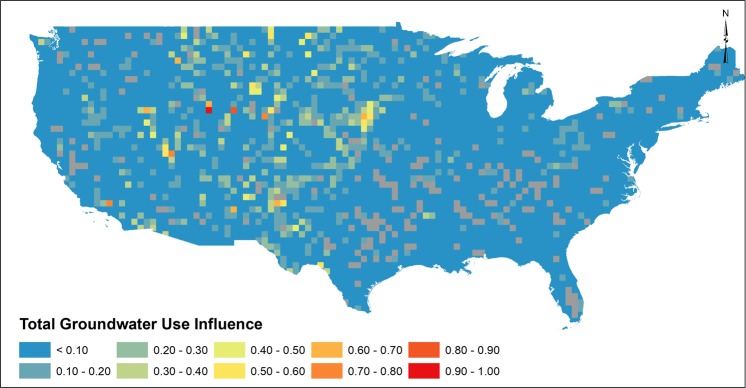
Influence of annual groundwater use to monthly GWA as determined by dominance analysis. Greyed squares represent regions that may be influenced by reservoirs.

Annual groundwater use interpolated from 5-year USGS reports^27–30^ are used to extract the influence of groundwater pumping to observed groundwater storage changes. In the upper Midwest and Rocky Mountain region, influence of groundwater use was found to be >0.50 in isolated regions (Fig. 4) that are spatially correlated to high reported groundwater use (See Supplemental). Throughout the remaining areas of the contiguous US, groundwater withdrawals appear to exhibit weak influence on monthly groundwater storage changes. Connections between groundwater use and depletion are common in the literature^5,19,22^, the results of which appear to conflict with results in Fig. 4 and with previous studies which identified strong influence of pumping as compared to climate in groundwater systems^59^. For the evaluation presented here, however, the time span of GRACE (2003–2015) captures only recent spans of groundwater pumping. Groundwater abstractions modify groundwater systems in convoluted ways as a function of time after a stress is initiated. Early time processes create a reduction in storage, whereby a cone of depression forms due to dewatering of the aquifer, to fulfill pumping volumes^60^. As time progresses, the cone of depression no longer changes, thus resulting in no observable change in groundwater storage. This quasi-equilibrium condition of the cone of depression is conditioned on horizontal flow in the aquifer (Dupuit flow) whereby capture^61^ fulfills pumping volumes. If pumping rates remain steady (or approximately steady) over time, no change in groundwater storage would be observed by GRACE as no dewatering of the aquifer in the near vicinity of pumping shall occur. Further, as explained by Alley and Konikow^62^, capture represented by changes in groundwater flow are not observed by GRACE.

## Discussion

Although trends in groundwater storage (Fig. 2) correspond spatially to regions of high groundwater pumping (See Supplemental), results depicted in Fig. 4 suggest that complex groundwater processes associated with long-term groundwater pumping may fail to be captured by GRACE. Groundwater recharge (Fig. 2) and precipitation (Fig. 3) exhibited higher influence in month-to-month groundwater changes where annual groundwater use exhibited lower influence. Interpretation of results is influenced by several factors. An assumption that groundwater pumping would result in an immediate change in storage is prescribed in Fig. 4, where in reality, changes in groundwater storage due to pumping depends on a variety of physical hydrogeologic characteristics (storativity, hydraulic conductivity, presence/absence of confining layers), aquifer geometry and groundwater/surface water interactions. Thus, local-scale physical properties exert important controls regarding any observable change in groundwater storage that may not be fully captured by GRACE. USGS water use reports are based on reported, estimated or calculated methods and, as such, it is impossible to estimate uncertainty of water use magnitudes. Minimal monitoring of private groundwater pumping^63^ further suggests that groundwater use magnitudes are likely underestimated. The approach using interpolated annual groundwater use records ignores annual variability in groundwater use, which may affect dominance analysis results depicting groundwater use influence in Fig. 4. Lastly, JPL GRACE mascon^64^ solutions at 3-degree native resolution may distort influence of local or regional changes in groundwater storage. Thus, the analysis presented in this study focused on regional observations of influence to satisfy recommended GRACE spatial resolution (>150,000 km^2^). Despite these constraints, however, our results corroborate recent studies that suggest groundwater storage changes are more influenced by climate^9,12,13^, whereby groundwater pumping rates increase over short time period to fulfill increased water demands during precipitation deficits. Short-term increases in pumping rates would cause a shift in the cone of depression, resulting in a decrease in groundwater storage that may be observed by GRACE. Results depicted in Figs 2–4 indicate groundwater storage changes as observed by GRACE are reflective of reduced recharge and increased pumping during periods of precipitation deficits and drought, which we define as climate-induced groundwater depletion. Thus, although long-term pumping causes shifts in sources of water pumped from the aquifer^65^, monthly groundwater storage changes are documented to be influenced by climate variability whereby increased groundwater pumping occurs to satisfy short-term water demands. Corroborative studies^9,12^ and heuristic observations^1,5,15,66^ have predicted such groundwater responses to climate, yet have not quantitatively assessed as climate-induced groundwater depletion as depicted in Figs 2–4 using GRACE or argue that climate variability responses in groundwater systems require long time periods^9,12^. Assessing influence of precipitation, groundwater recharge and anthropogenic pumping as related to changes in groundwater storage provides evidence of coupled human-natural systems under climate variability to aid in addressing key water resources challenges and sustainability assessments of groundwater resources. The link between precipitation deficit and pumping are characteristic of scenarios which have reportedly resulted in permanent loss of groundwater storage in the Central Valley Aquifer of California^34^, further supporting the need for adaptive management interventions to mitigate groundwater storage loss when faced with climate variability. Ultimately, our evaluation of climate-induced groundwater depletion holds important implications for interpretation of groundwater storage changes, especially when considering consequences of climate variability which may magnify drought severity and magnitude, further straining future groundwater resources^1,15^. Great uncertainty persists regarding future relations between groundwater storage and climate, yet results shown here illustrate that, at large scale, groundwater responds rapidly due to combined influence of climate variability and groundwater pumping. Results presented provide critical information in linking groundwater storage responses to climate forcing, information necessary to inform sustainable groundwater strategies.

## Methods and Data

### GRACE

Monthly GRACE^67^ gravity coefficients from the JPL-RL05.1 M mascon solutions^25,64^ available for the period 10/2003–12/2015 were used to isolate gridded groundwater storage changes. Groundwater storage anomalies were estimated using a mass balance approach whereby auxiliary data permit isolation of a groundwater storage signal from terrestrial water storage^68^. The approach requires the assumption that the terrestrial water storage anomaly (TWSA) is composed of anomaly changes in soil moisture (SMA), snow water equivalent (SWEA), surface water/reservoir storage (SWA) and groundwater (GWA) as in Eq. 1.

1 $$TWSA=GWA+SMA+SWEA+SWA$$

Groundwater storage anomalies were estimated by rearranging Eq. (1), while errors in the GWA time series were estimated by propagating TWSA, SMA, SWEA and SWA errors^68^. Average monthly model output from the NLDAS model framework^45^ were used including Moasic^69^, NOAH^70^ and VIC^44^ to remove soil moisture (water equivalent in 0–1 m soil layer thickness) and SWA, represented by gridded surface runoff, from TWSA. Data obtained from the Snow Data Assimilation System (SNODAS)^71^ were used to account for water equivalent changes in SWE. Daily SWE data were spatially interpolated (upscaled) to 0.5-degree resolution from its native 1 km resolution and temporally interpolated to extract monthly SWE. Given the recognized influence of reservoir storage in the isolation of GWS, 0.5-degree grids with large reservoirs extracted from National Atlas of the US records were not evaluated for the study and are represented as grey grids in Figs 1–4. Anomalies were estimated by removing the time series mean for the period 01/2004–12/2009 to be consistent with TWSA processing. Uncertainties in isolating groundwater storage from TWSA have been documented^17,72^ and are largely attributed to simulation of soil moisture in hydrologic models. Our approach in isolating a residual time series representing groundwater storage employs approaches commonly used in GRACE-derived groundwater storage studies^4,20,22,73^.

### Climate Variables

Monthly gridded climate data including monthly precipitation and monthly temperature (max, min and mean) were collected from the PRISM Climate Center Group^74^. Precipitation totals for 3-months and 12-months preceding a groundwater storage anomaly were evaluated in the analysis.

### Groundwater Use

Groundwater use data reported by the U.S. Geological Survey were used to characterize gridded groundwater extraction estimates and auxiliary data, including population served by groundwater and irrigated acres. USGS water use reports for 2000^27^, 2005^30^, 2010^29^ and 2015^28^ were used for the analysis. Annual records were estimated from 5-year increment datasets by temporal interpolation and spatially-weighted averaging was used to interpolate county-based values to 0.5-degree grids (See Supplemental).

### GWA Trend

Gridded groundwater trend magnitude and significance were estimated by the Seasonal Mann-Kendall trend test and the Sen Slope estimator to minimize the influence of nonnormally distributed variables in the analysis^26^. The Seasonal Mann-Kendall trend test computes traditional Mann-Kendall tests for each month (m = 12) and then combines information to assess a Kendall’s S^75^, in this case S_k_, given by

2 $${S}_{k}=\sum _{i=1}^{m}{S}_{month}$$

Where

3 $${S}_{month}=\sum _{i=1}^{n-1}\sum _{j=i+1}^{n}sgn({x}_{j}-{x}_{i})$$

In Eq. 3, x is the data point, groundwater storage in our case, at reference times i and j, while sgn(xj-xi) is 1 if xj > xi, −1 if xj < xi and 0 otherwise.

To estimate trend magnitude, the Sen Slope estimator (b) was applied where

4 $$b=median(\frac{{Y}_{j}-{Y}_{i}}{{X}_{j}-{X}_{i}})\forall \,j > i$$

In Eq. 4, Y represents monthly GWA and X represents the time of the observation^26^.

#### MRL

Ordinary least squares (OLS) multivariate regression (MLR) procedures were used to estimate model parameters for each 0.5-degree grid of the hybrid physical/statistical model. The proposed model took the general form of

5 $$GW{A}_{t}={\beta }_{0}+{\beta }_{1}{X}_{1}+\ldots +{\beta }_{n}{X}_{n}+{\varepsilon }_{t}$$

Where X_i_, …, X_n_ are explanatory variables (see Supplemental), $\beta \,$_0_, …, $\,\beta \,$_n_ are model coefficients and ${\varepsilon }_{t}$ are normally distributed model errors with zero mean and constant variance ${\sigma }^{2}\,$. A hybrid multivariate statistical/physical modeling approach was taken to account for primary determinants of groundwater storage changes given the groundwater continuity equation (see Supplemental for full list of potential explanatory variables). Backward and forward stepwise regression methods combined with randomized best subsets regression were employed to extract robust multivariate models at each 0.5-degree grid. Variable retention was carried out for variables that were significant (p < 0.05). To ensure that statistical influence was not compromised, performance diagnoses for normality of residuals (p < 0.10), variable independence measured as a variance inflation factor, multicollinearity, and homoscedasticity were tested. Model formulations with predicted R^2^ values greater than 0.70 were used for Figs 2–4. Grids with predicted R^2^ values of less than 0.70 were depicted as having an influence of zero for specific variables in Figs 2–4.

### Dominance Analysis

Dominance analysis approaches^76^ were applied to retrieve the notion of parameter influence on monthly GWA. In brief, dominance analysis computes the R^2^ for any predictor variable, in this case precipitation, groundwater recharge and total groundwater use, by evaluating conditional dominance of that variable for submodels (0 to p-1, where p represents the number of total submodels)^77^. Results depicted for groundwater recharge (Fig. 2), precipitation (Fig. 3) and total groundwater use (Fig. 4) were generally the most influential variables for all models tested. Often, models included other variables deemed significant although their influence was not discussed in this manuscript.

### Code Statement

General codes used in this study are available from the corresponding author upon request.

## Supplementary information

Supplementary Information for Identifying Climate-Induced Groundwater Depletion in GRACE Observations

## Data Availability

All data that support this work are publicly available from sources listed in the article, methods and supplemental material.
