# Supplementary material for: Identifying Climate-Induced Groundwater Depletion in GRACE Observations
Source: Sci Rep. 2019 Mar 11;9:4124. doi: 10.1038/s41598-019-40155-y (PMC6411996; doi:10.1038/s41598-019-40155-y)
Supplement: Supplementary file 1 — Supplementary Information for Identifying Climate-Induced Groundwater Depletion in GRACE Observations [file 41598_2019_40155_MOESM1_ESM.pdf]

Supplementary Information for

Identifying Climate-Induced Groundwater Depletion in GRACE Observations

Authors: Brian F. Thomas[1,*] and James S. Famiglietti[2]

Affiliations:[1] Department of Geology and Environmental Science, University of Pittsburgh, Pittsburgh PA, 15260, USA
[2] Global Institute for Water Security, School of Environment and Sustainability, and Department of Geography and Planning, University of Saskatchewan, Saskatoon, SK, S7N 0J9, Canada

Corresponding Author: Brian F. Thomas
Geology and Environmental Science
University of Pittsburgh
4107 O'Hara Street
Room 200 SRCC Building
Pittsburgh PA 15260
bfthomas@pitt.edu
+1 (412) 624-8780

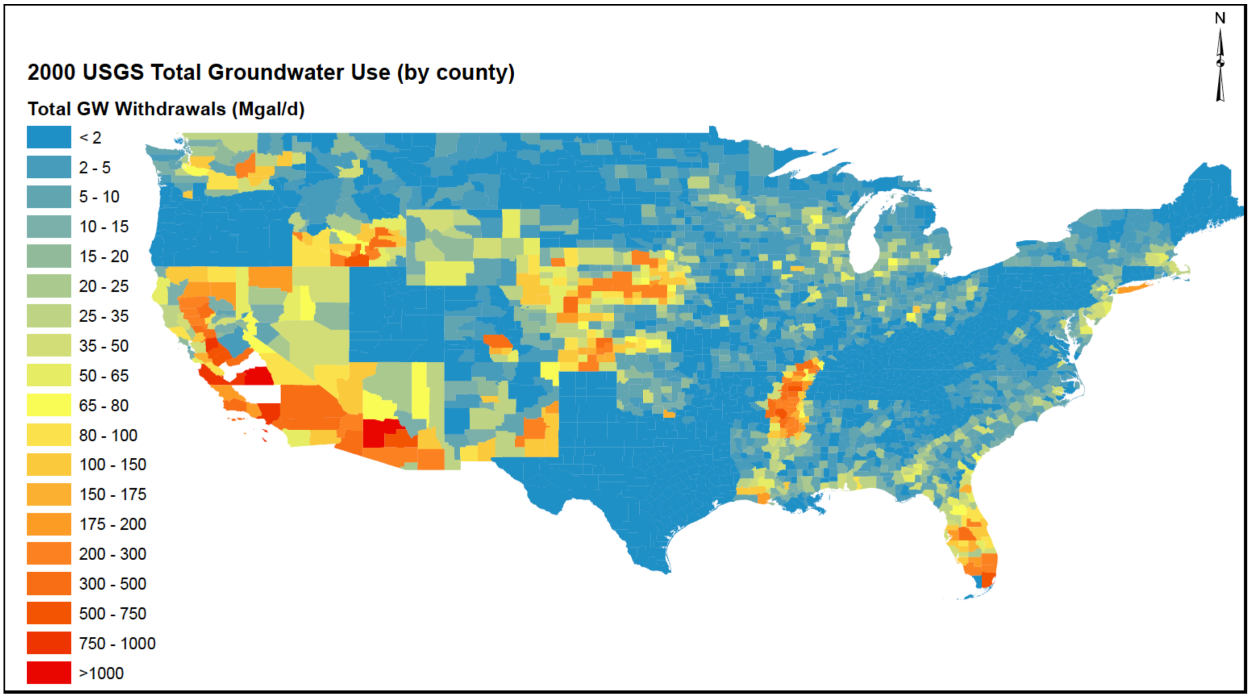

Supplementary Figure 1: County-based total groundwater use based USGS reports[1].

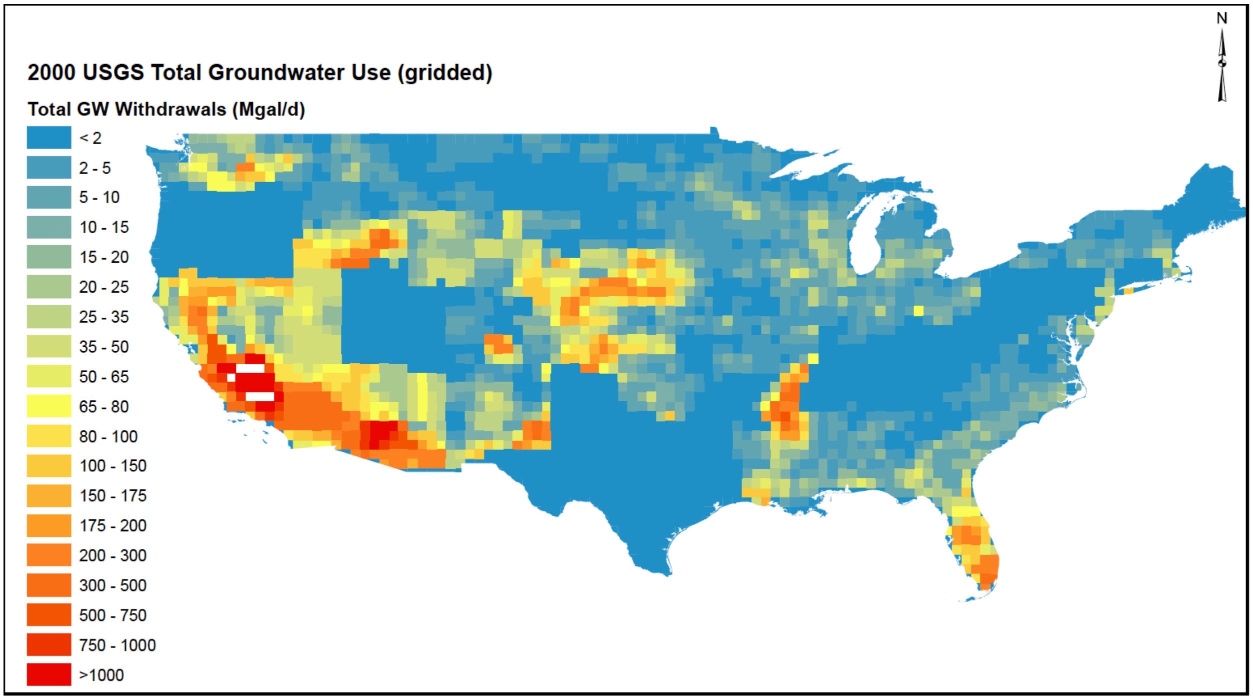

Supplementary Figure 2: 0.5-degree gridded USGS total groundwater use based on area-weighted averaging. Note states without data from the USGS databases.

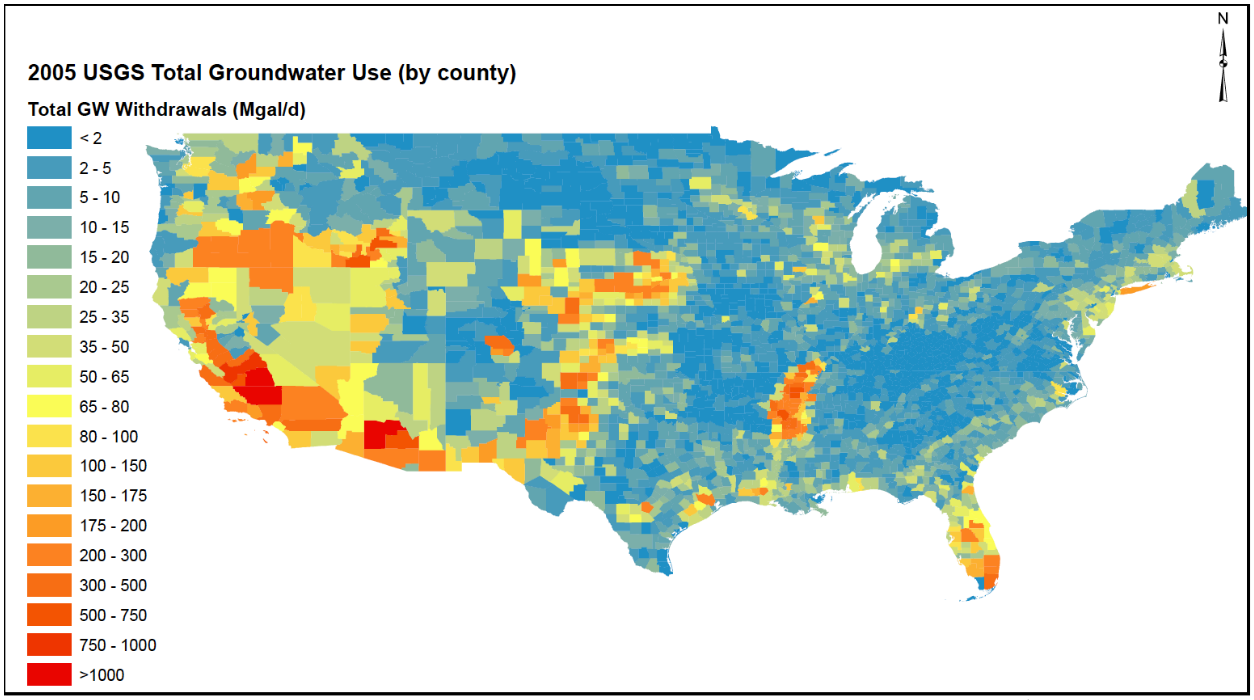

Supplementary Figure 3: County-based total groundwater use based USGS reports[2].

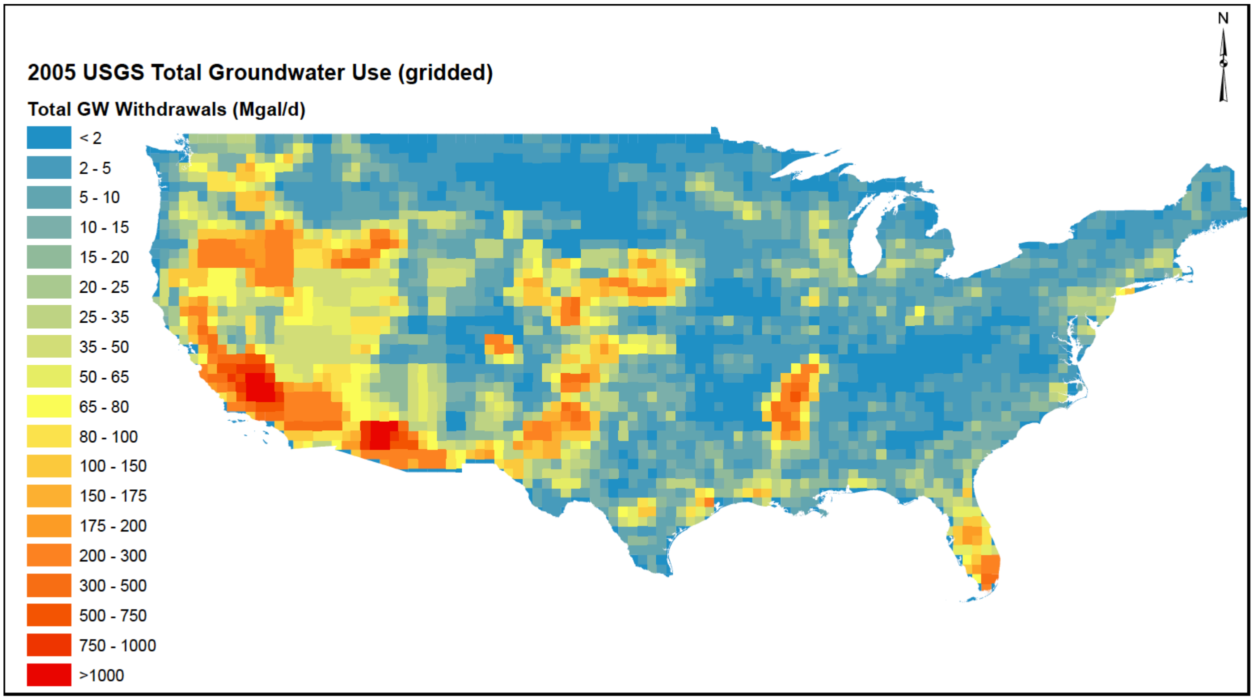

Supplementary Figure 4: 0.5-degree gridded USGS total groundwater use based on area-weighted averaging.

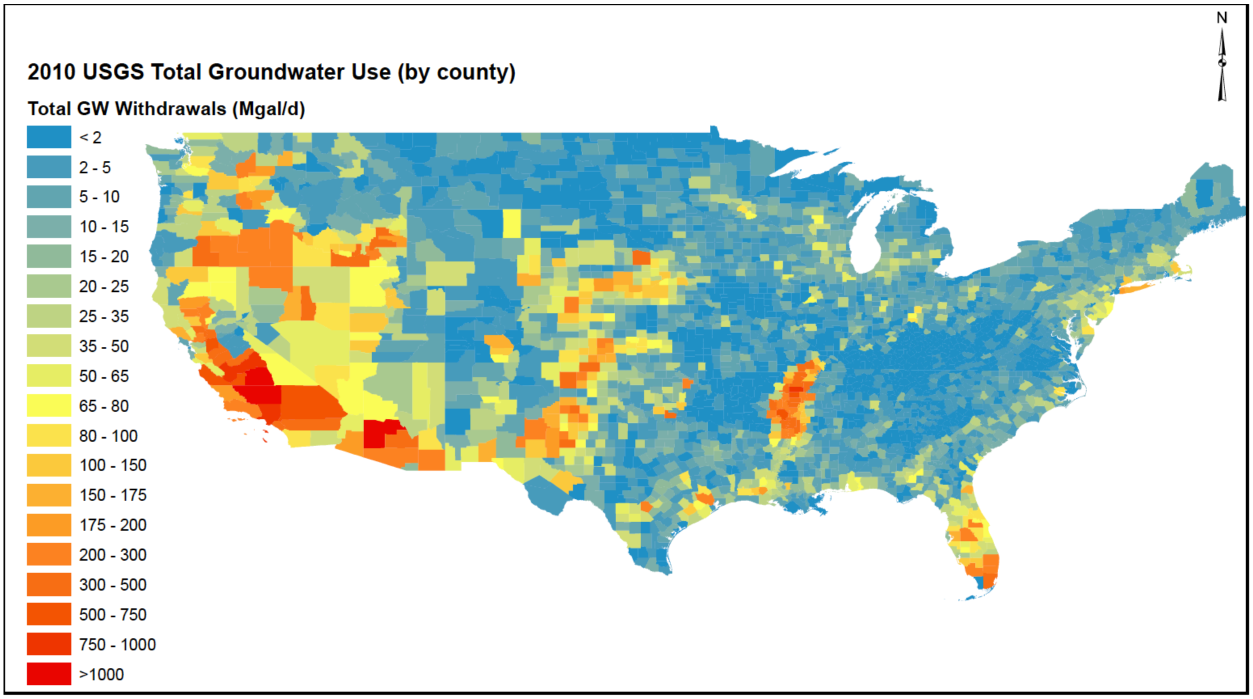

Supplementary Figure 5: County-based total groundwater use based USGS reports[3].

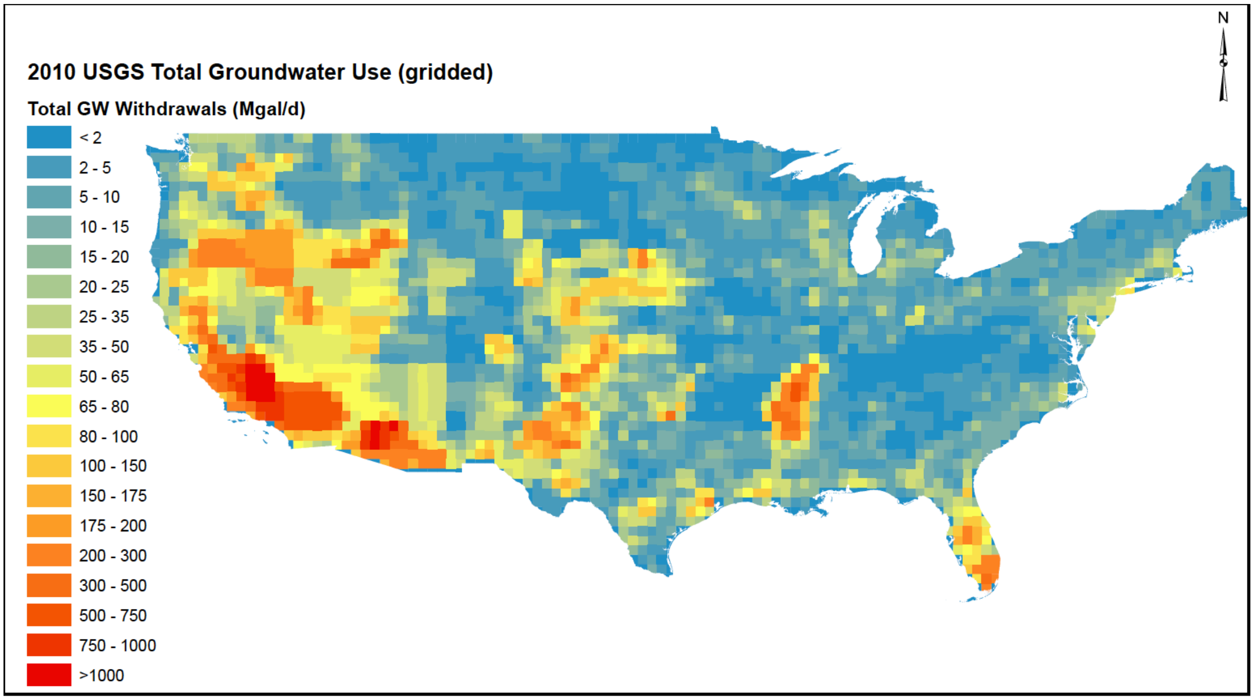

Supplementary Figure 6: 0.5-degree gridded USGS total groundwater use based on area-weighted averaging.

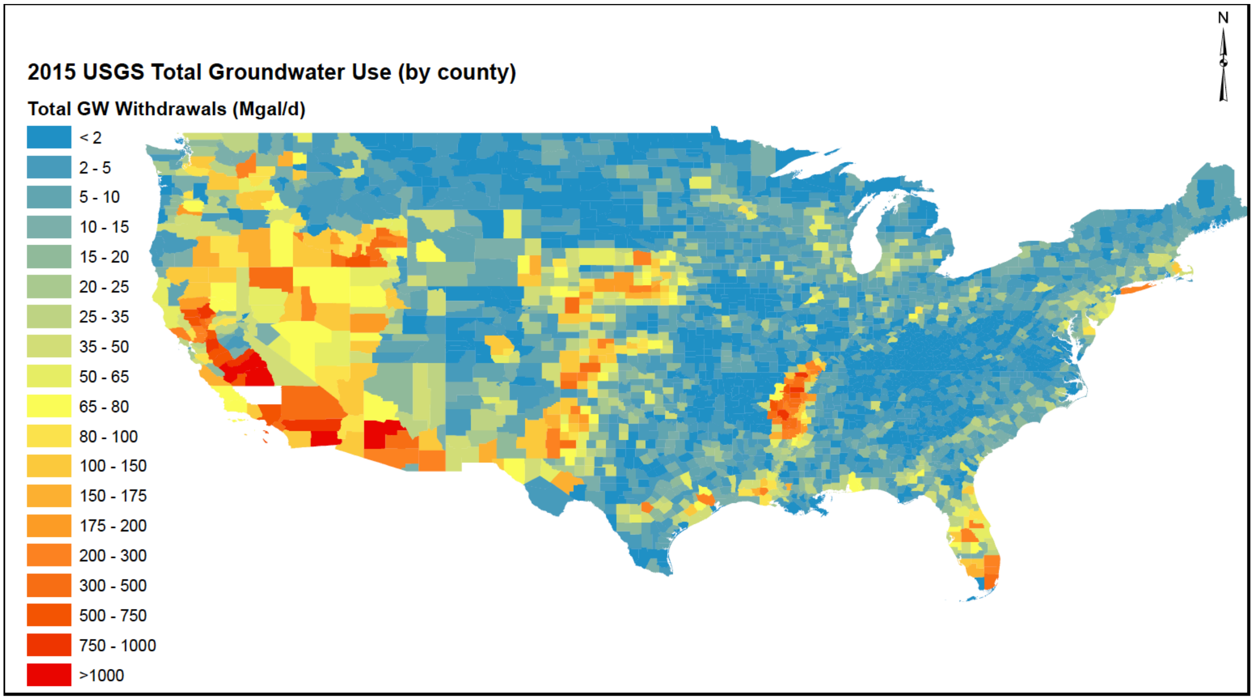

Supplementary Figure 7: County-based total groundwater use based USGS reports[4].

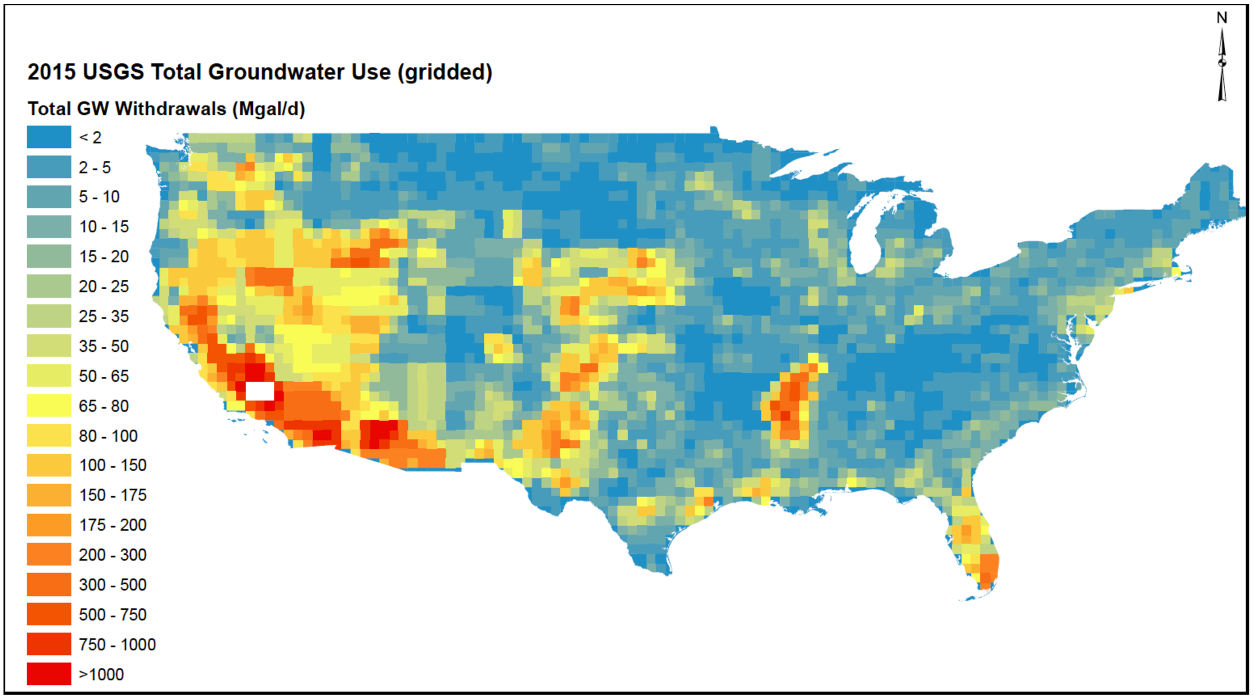

Supplementary Figure 8: 0.5-degree gridded USGS total groundwater use based on area-weighted averaging.

Supplementary Table 1: Explanatory variables tested for MLR procedures.

Variable Name	Description	Source
Pt	Monthly precipitation for time t	PRISM
P3t	Total 3-month preciptation preceeding time t	PRISM
P12t	Total 12-month preciptation preceeding time t	PRISM
Tmax_t	Maximum monthly temperature for time t	PRISM
Tmin_t	Minimum monthly temperature for time t	PRISM
Tmean_t	Mean monthly temperaure for time t	PRISM
GWRt	Monthly groundwater recharge for time t	NLDAS
GWR3t	Total 3-month groundwater recharge preceeding time t	NLDAS
GWR12t	Total 12-month groundwater recharge preceeding time t	NLDAS
SSPop	Population served by groundwater	USGS
DO.WGWFr	Domestic groundwater use	USGS
IN.WGW	Industrial groundwater use	USGS
IR.WGW	Irrigation groundwater use	USGS
TOT.WGW	Total groundwater use	USGS
TotPop	Total population	USGS
IR.IrTot	Total irrigated acres	USGS

1. Hutson, S. S. *et al.* Estimated Use of Water in the United States in 2000. in (US Geological Survey Circular 1268, 2004).
2. Kenny, J. F. *et al.* Estimated Use of Water in the United States in 2005. in 52 p. (U.S. Geological Survey Circular 1344, 2009).
3. Maupin, M. A. *et al.* USGS Circular 1405: Estimated Use of Water in the United States in 2010. *United States Geol. Surv.* (2010). doi:http://dx.doi.org/10.3133/cir1405.
4. Dieter, C. A. *et al.* *Estimated use of water in the United States in 2015*. (2018). doi:10.3133/cir1441
